# Results of the Voice Study: Stress and working conditions in the health system in a long-term comparison between occupational groups

**DOI:** 10.1192/j.eurpsy.2022.1278

**Published:** 2022-09-01

**Authors:** P.-S. Platzek, L. Jerg-Bretzke, Y. Erim, F. Geiser, P. Beschoner

**Affiliations:** 1 Ulm University Medical Center, Department Of Psychosomatic Medicine And Psychotherapy, Ulm, Germany; 2 University Hospital of Erlangen, Friedrich-Alexander University Erlangen-Nürnberg, Department Of Psychosomatic Medicine And Psychotherapy, Erlangen, Germany; 3 University Clinic of Bonn, Department Of Psychosomatic Medicine And Psychotherapy, Bonn, Germany

**Keywords:** Stress, Covid-19, pandemic, working conditions

## Abstract

**Introduction:**

Epidemics such as the Covid 19 pandemic in 2020/2021 increase the psychological stress among health-care workers (HCW) (cf. Mulfinger et al. 2020, da Silva et al. 2020).

**Objectives:**

The aim of this work is to investigate whether the stresses and working conditions have changed in the course of the pandemic and whether there are differences between different occupational groups in the health sector.

**Methods:**

In the first (T1) and second wave (T2) of the Covid 19 pandemic, the pandemic-specific working conditions and stresses were surveyed and analysed (descriptive, T-test, ANOVA) using 15 self-generated items on n=1036 HCWs and presented in a comparison of occupational groups.

**Results:**

Four occupational groups (doctors, nurses, medical-technical assistants, psychologists) were analysed: the highest stress was shown by the occupational group of nurses stress mean difference (MD) 0.453, p 0.000/working condition MD 0.993, p 0.000), the lowest by psychologists (stress MD 0.242, p 0.000/working condition MD 0.466, p 0.000). With regard to stress and working conditions, there was a significant difference between the two measurement points (p 0.000). However, no significant difference between T1 and T2 was found within the occupational groups.

**Conclusions:**

In summary, stress and the working conditions have changed over the long-term. This applies to all occupational groups; no significant difference can be detected within the groups. The results are in line with the infection pattern. The increase in stress and the deterioration of working conditions during the pandemic indicate that there is an urgent need for action to keep healthcare workers stable and healthy.

**Disclosure:**

No significant relationships.

